# A Reinforcement Learning Based Approach for Efficient Routing in Multi-FPGA Platforms

**DOI:** 10.3390/s25010042

**Published:** 2024-12-25

**Authors:** Umer Farooq, Habib Mehrez, Najam Ul Hasan

**Affiliations:** 1 School of Computer Science and Engineering, University of Sunderland, Sunderland SR6 0DD, UK; 2 LiP6 Laboratory, Sorbonne Universite, 75005 Paris, France; 3 School of Computing and Digital Technologies, Sheffield Hallam University, Sheffield S1 1WB, UK

**Keywords:** reinforcement learning, prototyping, multi-FPGA platforms, backend flow, inter-FPGA routing

## Abstract

Prototyping using multi-FPGA platforms is unique because of its use in real-world testing and cycle-accurate information on the design. However, this is a complex and time-consuming process with multiple sub-steps. Among its sub-steps, inter-FPGA routing is the one that can take a significant percentage of total prototyping time. The share of inter-FPGA routing is projected to increase further over time with the ever-increasing complexity of the target designs. In this work, we propose to integrate a Reinforcement Learning (RL)-based framework to speed up the inter-FPGA routing process. For this purpose, we first find a trade-off between the exploration and exploitation approach (also termed as the ϵ-greedy approach) in our RL-based framework while not affecting the final Quality of Results (QoR). To gauge its effectiveness, we then perform an extensive comparison between the proposed framework and established routing approaches. In this regard, a set of fourteen complex benchmarks is used, and the results of the proposed framework are compared against existing routability- and timing-driven routing approaches. Experimental results reveal that, on average, the proposed RL-based framework speeds up the inter-FPGA routing process by 45% and 32%, compared to routability- and timing-driven routing approaches, respectively. The speedup at the routing step further leads to an overall speedup of the backend flow by 22% and 15%, respectively.

## 1. Introduction

Owing to ever-improving design techniques and shrinking processing technologies, the computation capability of modern circuits has increased tremendously over the past few years. The increase in the computation power of modern day System on Schip (SoC) architectures is such that it can even eclipse the processing capabilities of the most powerful computers from a couple of decades ago [1]. It is common to have multiple Central Processing Units (CPUs) in modern day SoCs for general purpose computing. Their computing power is further enhanced with the addition of Graphic Processing Units (GPUs) [2]. The enhancement in the circuit capability has started a new computing era [3] and helped rapidly advance research in different domains like smart homes, fog computing in the Internet of Things (IoTs), autonomous vehicles, and Artificial Intelligence (AI), to name a few [4,5,6]. Specifically, if we take the example of the rapid deployment of AI in many modern day applications over the past couple of years, it can largely be attributed to three main enablers, namely better computation power of underlying circuits, availability of big data, and breakthrough in Deep Learning (DL) algorithms [7,8,9]. The growth in the computing power of digital circuits has been sustained for the past number of years, and it is forecasted to grow in the future with the advent of newer technologies [10].

The enhancement in the computing power of modern day digital circuits, however, has come at the cost of the increasingly complex design process, which is further exacerbated by mounting design costs, narrower time-to-market window, and shorter life cycles of these circuits [11]. Today, it is common to have millions of logic gates in an SoC with multiple power and clocking domains. Moreover, the costs associated with the design of new circuits can go up to millions of dollars and span anywhere from six months to two years. In this context, the verification of the circuit prior to fabrication (also termed as pre-silicon verification) and seamless chip supply to end users become a hugely important step as a rogue circuit or supply chain disruption [12] can cost a company a huge fortune and damage its reputation. Pre-silicon verification of a circuit is a critical as well as complex task, and it is becoming even more daunting with increasing circuit complexity and shrinking process technology. As per a survey conducted by Mentor Graphics in 2018, designers spend approximately 53% of their time on verification alone during the design cycle of a prototype circuit, and this time is projected to increase further with sub 5 nanometer circuits. This is because of the fact that over the past number of years, the complexity of circuits has been increasing at 58% per year, whereas the capability of design engineers increased only at 21% per year, and this gap is widening further with newer technologies [13].

Historically speaking, pre-silicon verification is performed using one of the four techniques, namely simulation, emulation, virtual prototyping, and Field Programmable Gate Array (FPGA)-based prototyping. Each of these techniques has its associated advantages and disadvantages. For example, simulation-based verification is used to check the hardware description of an SoC design. This technique offers complete system visibility with quick setup and inexpensive solutions [14]. However, this technique is rather slow, and it is not suitable for even moderately complex designs. In simulation-based verification, the behavior of the system is completely modeled through variables and configurations [11]. Compared to simulation, emulation-based verification completely mimics the hardware features of the system under consideration. The only difference is that emulation is performed in a virtual environment rather than the real world. Emulation offers faster speed, complete system visibility, and high scalability. Emulation-based verification solutions are offered by a number of vendors [15,16], but they are quite expensive and unaffordable for smaller companies and academia. Virtual prototyping is another verification technique [17] that is used to accelerate the design process and check the software model of a system. However, it is only a software verification technique and cannot be used for the cycle and bit-accurate checking of a digital system. Finally, we have FPGA-based prototyping [11], which is the only technique that gives the cycle and bit accurate [18] information of the design with real-world interfacing experience. Moreover, compared to emulation, it has a much smaller footprint. However, it has its own disadvantages as well. For example, it gives poor system visibility, requires expertise both in hardware and software, and is more expensive as compared to simulation-based techniques. A comparison of the four verification techniques is also given in Table 1.

Among the four verification techniques, FPGA-based prototyping is considered to be unique as it offers real-world testing experience along with hardware/software co-verification of the design under consideration. FPGAs have come a long way since they were first introduced as glue logic three decades ago. Modern day FPGAs are billion transistor circuits [19], and they have applications in areas like data centers, autonomous vehicles, and smart homes. Despite huge logic capability, the reconfigurable and generalized nature of FPGAs makes them much larger, slower, and more power hungry as compared to their Application Specific Integrated Circuit (ASIC) counterparts. There exists a huge gap between FPGAs and ASICs, and this is only going to get larger with increasing ASIC complexity and shrinking processing technology [20]. Because of this reason, multiple FPGAs are usually required to prototype even a moderately complex ASIC design, and their number can increase significantly with the complexity of the design under consideration [21]. Prototyping an ASIC design on a multi-FPGA platform is a daunting task as it requires expertise both in hardware and software. The efficiency and performance of the final prototype design are directly related to the quality of the backend flow tools of the multi-FPGA prototyping process.

The backend flow of multi-FPGA prototyping consists of multiple steps. It starts with the hardware description of the design, which is first synthesized. The design is next partitioned [22,23] where the principle constraint is to divide the design into equal parts with minimum communication interconnect between the partitioned parts. Another prominent constraint of the partitioning algorithm is that each partitioned part should not exceed the logic capacity of the target FPGA architecture. To satisfy these constraints, different partitioning approaches are proposed. For example, the authors in [24,25] use an analytical placement technique that gives good results for small designs with slight manual intervention. However, this technique does not guarantee consistent results, especially for large designs. Simulated annealing is another partitioning approach that is very suitable for mesh-based architectures [26,27]. This approach promises optimal solutions by minimizing the Manhattan distance between connected instances. The third partitioning approach is particularly useful for hierarchical designs, and it minimizes the number of signals traversing between different partitions by using a min-cut approach [28]. The min-cut approach can be applied to a design in a flat or multilevel manner where the multilevel approach [29] has been known to produce good results in a reasonable time.

Although partitioning algorithms try to keep the inter-partition communication to a minimum, the number of nets between the partitions is usually quite large as compared to the available physical lines between different FPGAs of multi-FPGA boards. This brings us to the problem of inter-FPGA routing, where the nets between the FPGA partitions (also termed as cut nets) are routed in a Time Division Multiplexed (TDM) manner. The number of cut nets passing between the FPGAs through a single line is termed as the multiplexing ratio. The objective of an inter-FPGA routing algorithm is to route cut nets in the shortest possible time while keeping the multiplexing ratio to a minimum. Once the inter-FPGA routing culminates, the partitioned design, along with routing info, is passed to the intra-FPGA placement and routing flow that culminates with in-circuit verification of the design.

In the multi-FPGA backend flow, partitioning and inter-FPGA routing are among the most critical steps, and the quality of these two steps plays a major role in the final prototype design. In particular, inter-FPGA routing is critical in the sense that it takes up to 40% of the total time spent in the FPGA backend flow, and this share is going to grow further in future [30]. The inter-FPGA routing is quite interesting and challenging in the sense that by the time a designer arrives at this step, the design is already partitioned, and the interconnect between different FPGAs is fixed. So, a routing algorithm has to use the available resources. Routing in FPGAs is considered to be an NP-complete problem [31] and it only gets harder to reach an optimal solution as the designs get more complex. In the past, different routing approaches have been used for FPGA routing. Some of the more commonly used techniques include obstacle avoidance [32], congestion avoidance [33], and negotiation-based, congestion-driven routing approach [34,35]. However, none promise to give optimal solutions, and all are based on heuristic approaches. The time required by heuristic algorithms to find a feasible solution increases exponentially with the complexity of the design under consideration, sometimes even rendering infeasible solutions as the complexity of the design inhibits a feasible solution in a reasonable time. The time taken and the quality of the solution produced by routing techniques are also affected by input parameters such as the cost function, the optimization approach, and the number of iterations. Recently, different researchers have used Machine Learning (ML) algorithms to automatically tune the parameters of FPGA backend flow and find a feasible solution in a short time [36,37]. However, these ML-based solutions are mono-FPGA oriented, and they focus particularly on the placement step of the backend flow. There are some other researchers who focus on the routing step as well [38,39,40,41]. A detailed discussion of this research is given in Section 2. However, it is important to mention here that almost all the aforecited work focuses on the single FPGA backend flow and no work has been performed in this regard on the inter-FPGA routing step of multi-FPGA backend flow.

In this work, we propose a novel, generic inter-FPGA routing approach where we use Reinforcement Learning (RL) to speed up the inter-FPGA routing process. For this purpose, we integrate RL into the existing inter-FPGA routing framework. For experimentation, we first explore the RL-based framework and determine the parameters that give the best results in terms of time and efficiency. Next, we compare the proposed approach against the existing congestion-driven routing approach. We compare the proposed approach against the routability- and timing-driven routing approaches. Our results show that the RL-based routing approach gives 45% and 32% better speedups against routability and timing-driven techniques, respectively, while giving similar or better Quality of Results (QoR). The contributions of this paper are summarized as follows:

- Integration of the RL-based approach in an existing inter-FPGA routing framework and exploration to find the best parameters through a large set of complex benchmarks.
- Speedup improvement in the inter-FPGA routing of multi-FPGA backend flow through the proposed RL-based framework.
- Performance comparison of the proposed approach against routability- and timing-driven variants of inter-FPGA routing through extensive experimentation.

In the rest of the paper, Section 2 gives a comprehensive overview of the existing state-of-the-art work, which is relevant to this paper. Section 3 gives an overview of different steps of multi-FPGA backend flow used. Section 4 then focuses on the inter-FPGA routing step of the backend flow and details the RL-based enhancements that we have integrated in the backend flow. Section 5 details the experimental results along with their analysis, and Section 6 finally concludes this paper with some discussion on future work.

## 2. Related Work

Inter-FPGA routing is one of the most critical steps of multi-FPGA backend flow. The quality of this step has a big impact on the performance of the final prototype design. In the past, the problem of inter-FPGA routing has been addressed using a number of techniques. For example, the authors in [32] use Integer Linear Programming (ILP) to solve the routing problem in a multi-FPGA context. This technique employs an obstacle avoidance approach where the nets and nodes, once used, are made unavailable for the rest of the nets. This kind of approach gives quick results for simple design. However, it renders infeasible results for complex problems and has the tendency to fall into local minima. The authors in [42] present a congestion avoidance inter-FPGA routing algorithm that avoids the local minima conundrum. However, their proposed approach takes more time as compared to the obstacle avoidance technique. The authors in [43] present a Pathfinder-based [34] inter-FPGA routing environment. However, this is mainly an exploration environment and it does not involve the usage of any AI or ML techniques.

In the past few years, a significant amount of research has been performed that uses AI or ML techniques to improve the Electronic Design Automation (EDA) in general and FPGA-based backend flow in particular. For example, the authors in [44] present an ML framework that automatically tunes parameters and finds a range that gives optimal results in a short time. The authors in [45] present another framework that makes use of ML and cloud-based computing techniques to help accelerate the FPGA-based design. Both of these works use a combination of ML algorithms like Support Vector Machines (SVMs), Bayesian Learning (BL), and Neural Networks (NN) to auto-tune FPGA backend flow parameters.

Apart from tuning parameters of the FPGA flow, there exists work that targets the optimization of individual steps of the flow. For example, the authors in [46,47] use Deep Neural Network (DNN) and Convolutional Neural Network (CNN) respectively to optimize the the synthesis of the design in an FPGA flow. Similarly, the authors in [36,37] optimize the placement step using ML framework and further use the placement information to predict the routing outcome of the design as well [48]. Recently, RL, which is a type of ML, has seen popularity in EDA because of its superior performance compared to other ML techniques [49,50]. The authors in [51] use an RL-based framework to speed up the placement step while giving similar or better QoR compared to existing heuristic techniques like simulated annealing [26]. Similarly, there is work [38] that uses ML techniques to predict the routability of the design under consideration. However, they do not use ML techniques to perform the detailed routing. The authors in [52] present another RL-based routing framework that speeds up the routing step while giving similar or better results compared to the existing routing techniques.

The discussion provided above suggests that lately, a significant amount of work has been performed to improve the FPGA backend flow. Some of the aforecited work performs optimization at the global level and uses ML techniques to tune the FPGA backend flow parameters. Some other work focuses on the individual steps of flow, like synthesis, placement, and routing. It is important to mention here that all this work focuses on single FPGA flow, and to the best of our knowledge, no work has addressed the multi-FPGA backend flow. In this work, we propose to use an RL-based framework that speeds up the inter-FPGA routing step while giving similar or better results compared to the existing solutions used for inter-FPGA routing. For experimentation, we integrate our proposed RL-based framework into an existing multi-FPGA exploration environment [21] and compare our results against routability- and timing-driven routing approaches. Our experimental results show that the proposed framework gives 45%, 32% better performance in terms of speed up while giving similar and sometimes surpassing QoR given by the existing framework. In the next section, we give a brief yet comprehensive overview of the multi-FPGA prototyping flow, and then we give details of our proposed enhancement in Section 4.

## 3. Multi-FPGA Backend Flow

**Figure 1:**
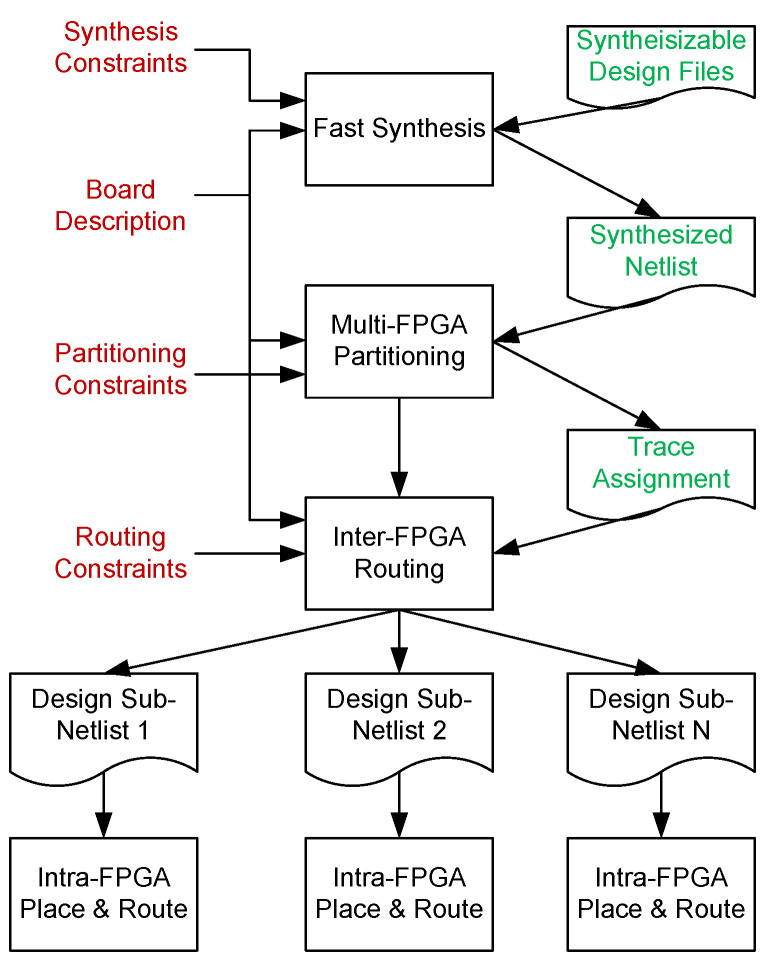
An overview of the multi-FPGA backend flow.

In this section, we give a brief description of the multi-FPGA flow used in this work. The overview of different steps of multi-FPGA backend flow is shown in Figure 1. It can be seen from the figure that the flow starts with the synthesis of the design, followed by partitioning, routing, and finally culminating at intra-FPGA placement and routing of the design.

### 3.1. Design Synthesis

**Figure 2:**
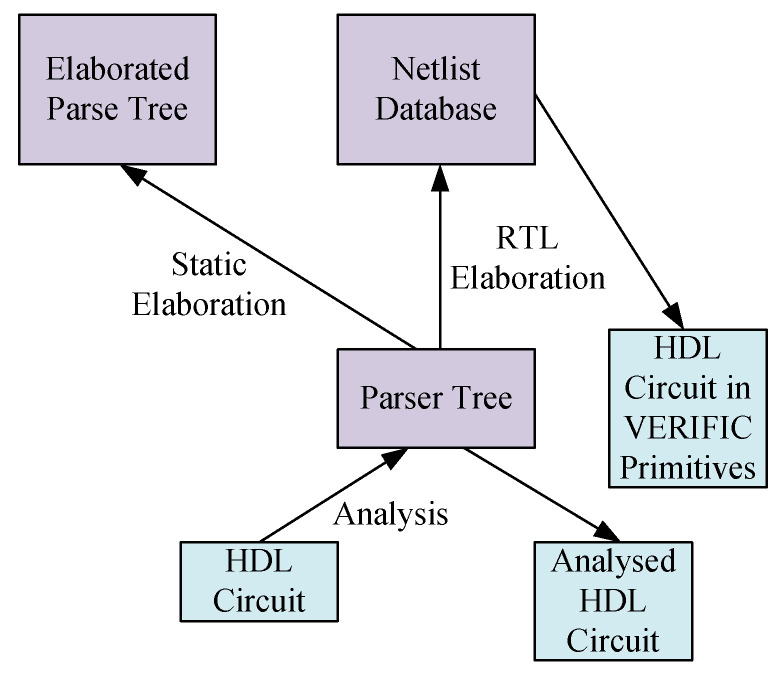
Design manipulation performed by VERIFIC.

Synthesis is a process where a design is logically optimized, and its hardware description is mapped to the library of the target architecture. In this work, we use the Design Space Exploration (DSE) tool [53] to generate the hardware description of the Multi Processor System on Chip (MPSoC) architecture. The MPSoC architectures generated through this tool vary in size and complexity. Some of them are mono-cluster, while others are multi-cluster. Apart from various co-processors, the hardware description of these MPSoCs also contains various complex components like RAM, ROM, and FIFOs, and the interconnect between these components is ensured through DSPIN Network on Chip (NoC) architecture [54]. The synthesis of the design is performed using the VERIFIC tool [55]. It is a very powerful parsing tool that gives complete information about the hierarchy of the design and transforms it into standard logic gate primitives. An overview of the manipulation performed by these tools is also shown in Figure 2. The parse tree function of the tool helps in analyzing the whole circuit and gives complete information about the interconnect of the design. This information is used by the partitioning step, which is described next.

### 3.2. Partitioning

**Figure 3:**
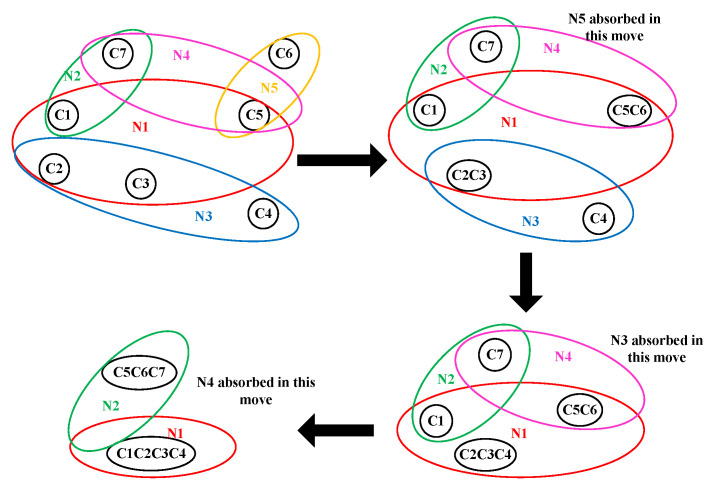
Clustering phase in partitioning.

**Figure 4:**
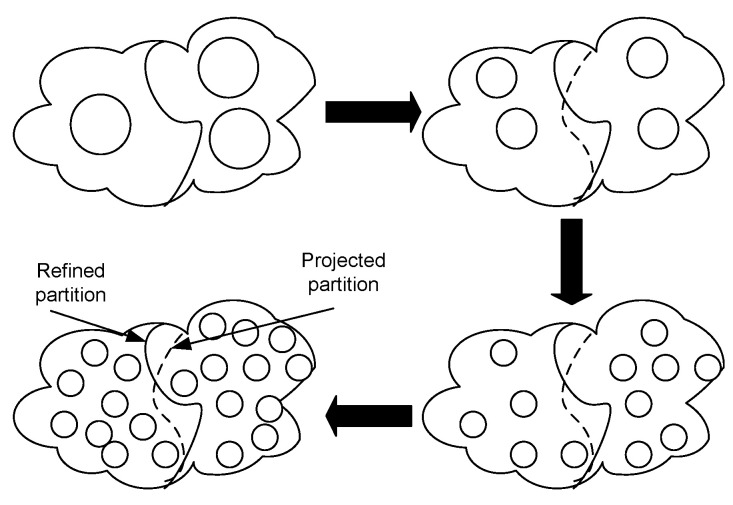
Refinement phase in partitioning.

Once the design is synthesized, the netlist is then partitioned using a multi-FPGA partitioning process. This is an important step, and the impact of a mal-partitioned design at this step cannot be undone at a later stage of inter-FPGA routing. Usually, a partitioning algorithm has to satisfy conflicting requirements of fitting the design to the logic capacity of the target architecture and minimizing the cut-net count. Finding an optimal partitioning solution for a given design is difficult [56]. In this work, we use heuristic hierarchical and multilevel partitioning approaches [57,58]. The hierarchical approach is more suitable for designs that have an inherent hierarchy in them, whereas a multilevel approach is better for designs exhibiting rather mesh-like interconnect. The graphical description of clustering and refinement steps involved in multilevel partitioning is shown in Figure 3 and Figure 4 respectively. During clustering, based on connectivity, smaller instances are combined to make bigger clusters. This process is repeated over multiple levels until only a few clusters are left. Figure 3 pictorially explains the clustering process where a hypergraph of seven instances is converted into only two clusters after multiple iterations of clustering. In the next phase, refinement is performed where clusters are expanded in the reverse order, and instances are moved between the clusters to minimize the cut-net count. In this work, we have a complete overview of the design interconnect at the synthesis step, and based on that, we apply a partitioning approach (either hierarchical or multilevel) that better suits their interconnect. Because of the scope constraint, we have provided only a brief overview of the two partitioning approaches in this work. A detailed discussion of these partitioning approaches can be found in [58]. Once the design is partitioned, it is passed onto the inter-FPGA routing process, whose details are provided next.

### 3.3. Inter-FPGA Routing

**Figure 5:**
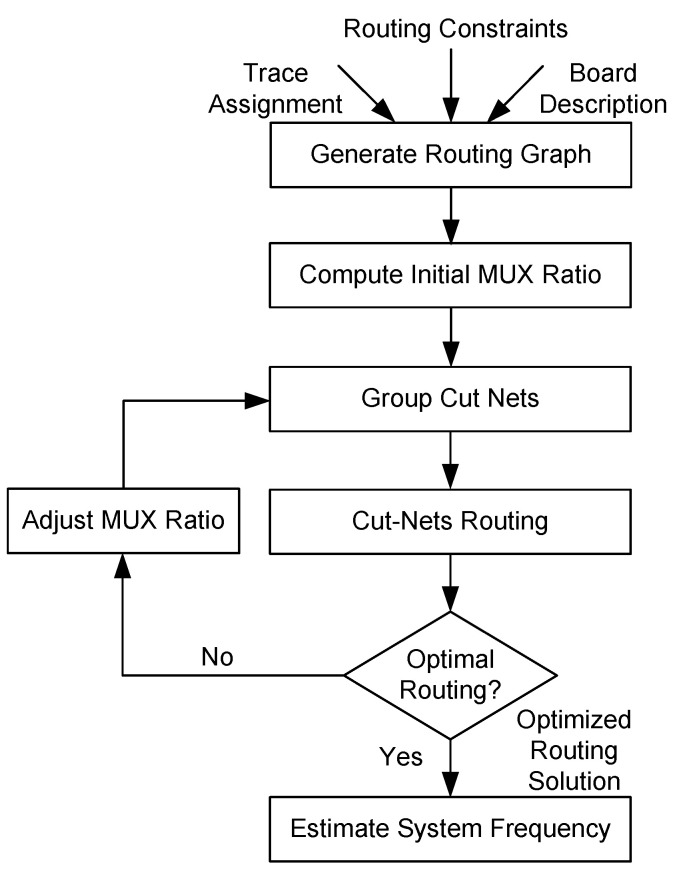
Inter-FPGA routing flow.

As discussed in Section 1, there is a huge disparity between the logic resources and the number of I/Os of modern FPGAs. Because of this reason, the number of cut nets between the partitions is way more than the available physical resources between the FPGAs. These cut nets are usually either single-source, single-destination (also termed as biterminal cut nets), or single-source, multi-destination (termed as multiterminal cut nets). The objective of a routing algorithm is to route these cut nets on the limited physical resources in a time division multiplexed manner while keeping the number of hops and multiplexing ratio to a minimum. An overview of the inter-FPGA routing process used in this work is shown in Figure 5. As a first step, information on multi-FPGA board is transformed into a routing graph G(V,E) where vertices ‘*V*’ correspond to the FPGAs and edges ‘*E*’ correspond to the connections between them. The information taken from the routing graph is next used to compute the initial mux ratio. The mux ratio is next used to group cut nets having the same source and destination. Cut nets are routed using the routing approach under consideration. This is the step where the most amount of time and computing resources are consumed. A detailed discussion on cut-net routing and proposed enhancements in this step is presented in Section 4.

Once all the cut nets are routed, the mux ratio is reduced through an iterative optimization procedure. This process continues until the best mux ratio with minimum hops is found, and the routing process terminates with the estimation of the execution frequency of the target design. After the inter-FPGA routing, the design subnetlists are passed through the vendor-specific flow to perform intra-FPGA placement and routing. The multi-FPGA backend flow culminates when the bitstreams of design are downloaded onto respective FPGAs, and in-circuit verification of the design is performed.

## 4. Proposed Enhancement

Inter-FPGA routing has been previously performed using different methods like ILP [32], congestion avoidance [42], and negotiation-based [43] routing algorithm, to name a few. In this work, we propose to integrate an RL-based framework into the inter-FPGA routing environment and evaluate its impact on the routing QoR. In this section, we give an overview of RL and also detail the proposed enhancement.

**Figure 6:**
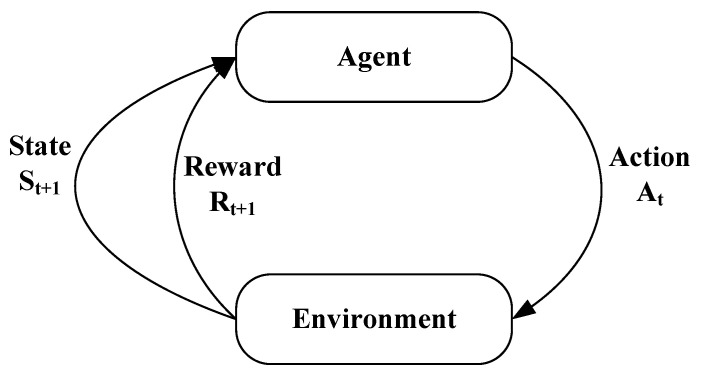
A sample reinforcement learning problem.

Recently, RL, which is an ML-based technique, has seen its applications in EDA [50]. An RL-based framework finds the solution to the problem at hand by taking actions and then learning from the consequences of those actions. A typical RL-based framework is shown in Figure 6. In an RL-based framework, at a given time *t*, an action $A_{t}$ is taken, and as a result of that, a reward $R_{t+1}$ is generated, and the system moves from the current state $S_{t}$ to the next state $S_{t+1}$. In this way, multiple actions are taken over time, and a log of those actions and their rewards is maintained. Over time, the new actions taken are affected by previously taken actions and their respective rewards. The pseudocode of RL is shown in Algorithm 1, where the outer loop iterates for a fixed number of iterations and the inner loop iteratively changes the state and reward values. The α, γ and ϵ are hyperparameters that control the learning, discounting, and exploration/exploitation balance of the algorithm. With each iteration of the outer loop, the ϵ_decay parameter gradually moves the algorithm away from exploration toward exploitation.
**Algorithm 1: **Pseudocode for RL-based framework
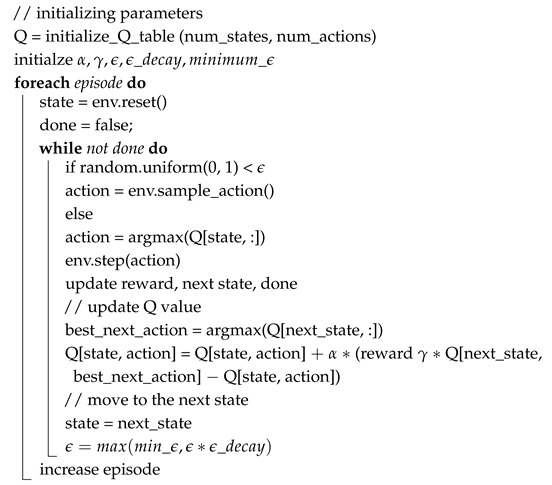


For the RL-driven approach, after experimentation and exploration, we set ϵ = 0.01 (tradeoff between exploration and exploitation) and γ = 0.1 (meaning the most weightage is given to the most recent 10% moves). The value of α is calculated using Equation (1) [52], where *M* corresponds to the number of iterations. The reward function of the RL-based framework is very important as it decides the future moves. For the RL-based framework, a basic reward function is usually used. The basic reward function is given in Equation (2) [52]. Although this function satisfies our objective, it has the tendency to fall in local minima as it penalizes the moves that result in an increase in conflicts. Hence, we modify the reward function to Equation (3). This reward function favors a more exploratory approach and avoids local minima by not penalizing the moves that cause an increase in conflict count.

(1) $$\alpha =1-e^{log(\gamma )/M}$$

(2) $$r_{t}=-\Delta_{conflict}$$

(3) $$r_{t}=\left\{\begin{array}{cc} -\Delta_{conflict}, & \mathrm{if}\;\Delta_{conflict}<0\\ 0, & \mathrm{otherwise}. \end{array}\right.$$

As discussed before, for inter-FPGA routing, a negotiation-based congestion-driven routing algorithm has been previously used. In this work, we compare routability- and timing-driven approaches of the negotiation-based algorithm against the RL-based framework. The pseudocode of the negotiation-based algorithm is shown in Algorithm 2. It can be seen from this code that this is an iterative algorithm where congested nets are routed, and their cost can be controlled by either Equation (4) or (5) [21]. The congestion cost of Equation (4) is applicable if the routability-driven approach is employed and that of Equation (5) is applicable if the approach is timing-driven. It can be seen from Equation (4) that the net cost is purely driven by present $p_{n}$ and historical $h_{n}$ congestion of the node. However, the cost function of Equation (5) indicates that the first part of the cost is dictated by the delay, and the second part is dominated by the congestion of the node. The criticality of the net for the timing-driven approach is calculated using the formula in Equation (6) [21], where slack(i,j) is the delay that could be added before it affects the critical path delay of the circuit while Dmax is the critical path delay.

(4) $$c_{n}=\left(b_{n}+h_{n}\right)\times p_{n}$$

(5) $$c_{n}=Crit\left(i,j\right)\times delay\left(n\right)+\left[1-Crit\left(i,j\right)\right]\times \left(b_{n}+h_{n}\right)\times p_{n}$$

(6) $$Crit\left(i,j\right)=1-\frac{slack(i,j)}{Dmax}$$

In our proposed RL-based framework, we use the state and reward function of Algorithm 1, which replaces the cost functions of Equations (4) and (5). The proposed RL-based approach is particularly interesting in the sense that it keeps track of fewer congestion parameters as compared to routability- and timing-driven approaches. In this approach, a single record of node congestion is maintained, and it helps in finding a conflict-free solution in significantly less time and iterations as compared to existing approaches.
**Algorithm 2: **Pseudocode of Pathfinder routing algorithm
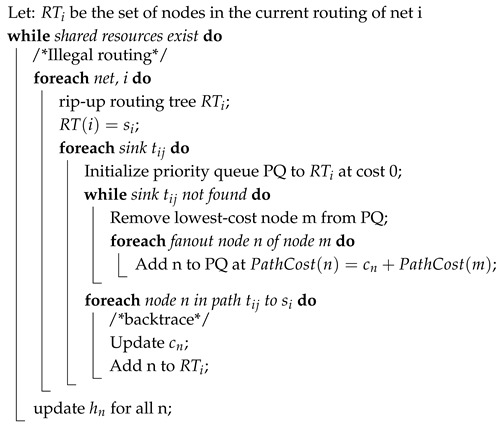


## 5. Results and Discussion

In this section, we present the experimental results obtained through the flow described in Section 3. The proposed enhancements discussed in Section 4 are integrated into the inter-FPGA routing step, and the comparison between routability-, timing-, and RL-based approaches is presented in this section.

### 5.1. Benchmarks

To perform experimentation and comparison between different routing approaches, we use a set of fourteen benchmarks that are generated through DSE tool [53] described in Section 3.1. Details of the benchmarks used in the experimentation are presented in Table 2. It can be seen from this table that we use two types of benchmarks for experimentation. Mono-cluster benchmarks are single-cluster, multicore benchmarks, and multi-cluster benchmarks are multi-cluster, multicore benchmarks. Both mono- and multi-cluster benchmarks have other components like RAMs, FIFOs, and UARTs etc. However, the interconnect structure of mono-cluster benchmarks uses the VCI network, whereas the interconnect structure of multi-cluster benchmarks is based on DSPIN NoC architecture [54]. The DSPIN network uses a mesh-based approach where each node in the mesh defines its cluster and interconnect. The mesh-based nature of DSPIN interconnect makes it completely scalable.

### 5.2. Results and Analysis

#### 5.2.1. Exploration Results

As discussed in Section 3, partitioning is an important step, which is followed by inter-FPGA routing. In this work, to best exploit the inherent characteristics of the benchmark interconnect, we employ two partitioning approaches. For mono-cluster benchmarks, we use a multilevel partitioning approach as it better optimizes the cut-net count for benchmarks with mesh-like interconnect. On the other hand, for multi-cluster benchmarks, we use a hierarchical partitioning approach as it better exploits the hierarchical interconnect between the clusters [21]. The partitioning results of two partitioning approaches are presented in Table 3. In this table, each benchmark has two types of cut nets: biterminal nets represent point-to-point interconnect, whereas multiterminal net count represents single source multi-destination net count. It is important to mention here that the multilevel approach is more time-consuming than the hierarchical approach. However, in this work, we use this approach only for smaller mono-cluster benchmarks where the gap between the two approaches is not significant, and it does not have an adverse impact on the overall execution time of the prototyping flow. Moreover, the focus of this work is efficiency improvement through routing, and we intend to explore the partitioning aspect of the flow in future work.

**Figure 7:**
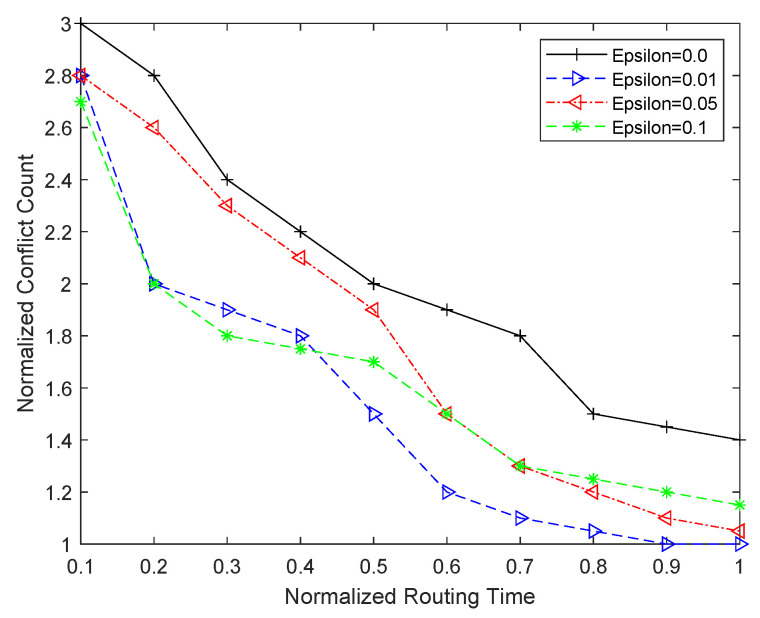
Routing time and conflict count comparison for different values of ϵ.

Before comparing different routing approaches, we first explore the best routing parameters for RL-based routing. For this purpose, we explore the impact of varying ϵ on the routing time and the results are shown in Figure 7. Our experimentation and exploration reveal that RL-based routing with either a purely greedy (i.e., ϵ=0) or purely exploratory (i.e., ϵ=1) approach does not give good routing results. It is clear from this figure that a purely greedy approach has the tendency to fall in a local minima. As a result of this pitfall, it does not find a conflict free solution in a given amount of time. For more complex benchmarks, this approach is unable to find the conflict-free solution in a reasonable time. On the other hand, a purely exploratory approach (i.e., ϵ=1) tends to spend too much time in exploration and does not necessarily improve the QoR. Our experimentation further reveals that an increase in value of ϵ to 0.01 gives a better trade-off between routing time and conflict count. This is mainly because of better hill climbing combined with slight exploration. However, a further increase in ϵ value does not necessarily further improve the QoR. For example, when ϵ is increased to 0.05 or 0.1, it increases the exploration but does not improve the convergence time. The impact of different values of ϵ on the quality of results is shown in Figure 7. It is clear from this figure that ϵ=0.01 gives the best results in the shortest time, and we use this value for the rest of the comparison with other routing techniques (i.e., routability- and timing-driven) under consideration.

#### 5.2.2. Comparison Results

In this work, we compare four different routing approaches and the mux ratio results of these approaches are shown in Table 4. In this table, ‘RD’ and ‘TD’ are routability- and timing-driven routing approaches. Both are congestion-driven, negotiation-based approaches. They are based on Algorithm 2, and their cost functions use Equations (4) and (5), which have already been discussed in Section 4. The ‘RLM’ and ‘RLNM’ columns in Table 4 represent routing results of RL-driven routing that uses Algorithm 1. The ‘RLM’ is the approach with memory (i.e., γ=0.1 where the most recent 10% moves are given maximum weight), and ‘RLNM’ is the approach without memory (i.e., γ=0, where no record of previous moves is maintained) respectively. It can be seen from this table that the RLNM routing approach gives the worst mux ratio results. This is because of the reason that this approach does not give any weight to the most recent moves. On the other hand, the RLM approach gives the maximum weight to its most recent 10% moves and adjusts its reward and next states based on the outcome of the moves carried out in the recent past. As far as the comparison between RD and TD is concerned, both approaches have historically been known to produce similar quality results in terms of mux ratio [43]. However, it is the time taken by the two approaches that makes the difference. Further discussion on this aspect is provided next.

The mux ratio results of Table 4 are used to calculate the execution frequency of each benchmark. The frequency comparison results are shown in Table 5. The frequency of individual benchmarks is calculated using Equation (7) [59]. The results of Table 5 give similar trend as observed in Table 4. The reason for this trend is that frequency results are largely dependent on the multiplexing ratio result. It can be seen from these results that the RLNM approach gives the worst overall results, and the TD and RLM approaches give comparable frequency results. It can be concluded from these results that from a mux ratio and frequency perspective, the RL-driven routing approach does not bring much improvement in QoR.

(7) $$sys\_freq=\frac{if\_freq}{mux\_ratio+hops+3}MHz$$

**Figure 8:**
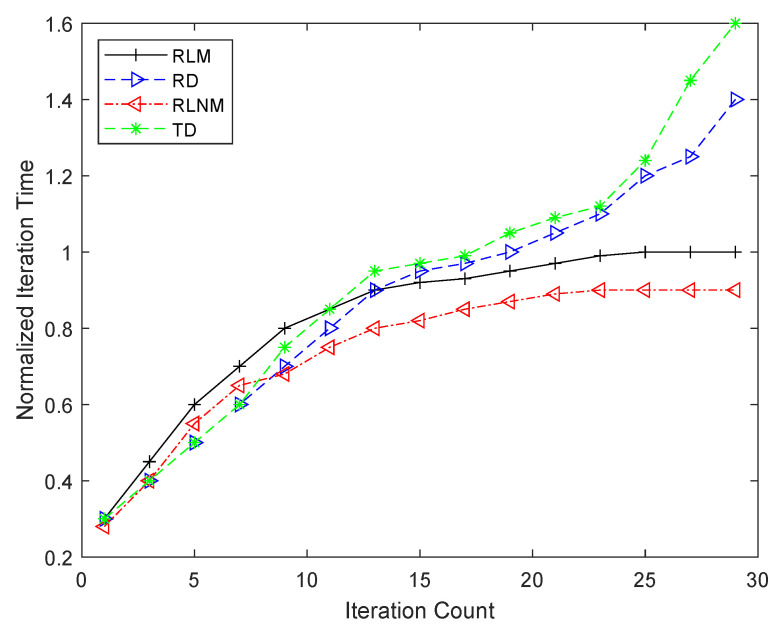
Iteration count and time taken per iteration comparison between four routing approaches.

As discussed in Section 3.3, inter-FPGA routing is an iterative process where the routing algorithm tries to find a conflict-free solution over multiple iterations. To have further insight into the routing results, we compare the iteration count and time taken by each iteration for different routing approaches under consideration. The results of this comparison are shown in Figure 8. It can be seen from this figure that for almost all four approaches, with an increase in iteration count, the time per iteration initially increases linearly. The reason for this increase is that with every passing iteration, the cost of congested nodes increases, and the routing algorithm has to spend more time to find the conflict-free nodes. However, beyond a certain number of iterations, the per iteration time taken by both RD and TD starts to increase exponentially, with TD giving particularly poor results. Moreover, it is important to note here that RLNM gives the best results because of limited exploration, and the RLM approach gives the best trade-off between RLNM and TD approaches. Furthermore, it is pertinent to mention here that for RLNM approach, spending less time per iteration does not necessarily translate into good QoR as evidenced in Table 5. This is because of the fact that there is no record of recent moves in the RLNM approach. It leads to limited exploration time and ultimately results in poor mux ratio and frequency results.

**Figure 9:**
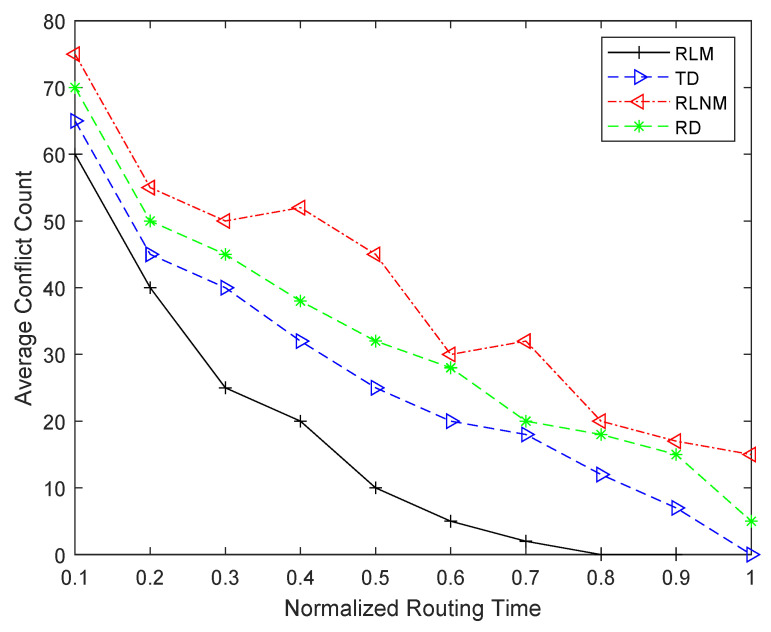
Routing time vs. conflict count comparison results for different routing approaches.

Finally, the comparison results of the average time taken by each routing approach to find a conflict-free solution is shown in Figure 9. It can be seen from this figure that, on average, the RLM routing approach gives the best results in terms of the time taken to reach a conflict-free solution. Although the RLNM approach requires less time per iteration, it requires significantly more iterations to find a conflict-free solution, which leads to the worst results in terms of the time taken to find a conflict-free solution. Moreover, it is clear from this figure that as compared to the RD routing approach, TD finds a conflict-free solution in less time despite spending more time per iteration. This is because of the fact that TD requires an overall smaller number of iterations compared to the RD approach.

It is clear from results presented in Table 4 and Table 5 and Figure 8 and Figure 9 that RL-based routing approach (i.e., RLM) either offers no or very small improvement in the QoR in terms of the frequency of the prototype design. However, it significantly reduces the time required to perform inter-FPGA routing while giving similar and, in some cases, better frequency results. Against Rd, TD routing approaches, for different benchmarks of Table 2, the individual time gain provided by the RLM approach in inter-FPGA routing varies between 27 and 45%. When compared to congestion-based, routability-, timing-driven (i.e., RD, TD) routing approaches, on average, the RLM approach gives 45% and 32% gains. As discussed in Section 1, inter-FPGA routing is one of the most time consuming steps of multi-FPGA prototyping flow. So, the gain obtained at this step through the proposed RLM approach translates into 22% and 15% reductions in total flow time compared to the RD and TD routing approaches.

## 6. Conclusions

Prototyping using multiple FPGAs is a challenging task that requires expertise at both the hardware and software levels. Routing is one of the most complex and time-consuming steps of mutli-FPGA-based prototyping. In this work, we propose an RL-based framework to speed up the inter-FPGA routing in particular and the overall backend flow in general. Through exploration, we find a fine balance between the exploration and exploitation approach of the RL framework, which gives good routing results in a reasonable time. We then evaluate the proposed framework by comparing its results against routability- and timing-driven, negotiation-based routing approaches. Our comparison results reveal that in terms of frequency, the RL-based framework gives almost similar results against the timing-driven approach. However, it is the average time taken per iteration and the overall time required to reach a conflict-free solution where we achieve significant gains. Our results show that the proposed framework requires, on average, 32% less routing time while achieving similar QoR, compared to the best congestion-driven routing approach.

In this work, we have focused on the speedup of the inter-FPGA routing process. In the future, we would like to integrate machine learning techniques at the synthesis and partitioning steps of the backend flow. We would be keen to evaluate the impact of machine learning techniques on the quality of results of individual steps and the speedup that we can achieve in the overall backend flow.

## Figures and Tables

**Table 1:** Comparison between four pre-silicon verification techniques.

Technique	Setup Time	Cost	Execution Speed	Footprint	Verification Type	Visibility
Simulation [14]	Small	Few thousand dollars	Few KHz	N/A	Hardware	Up to block level
Emulation [15]	Medium	Million dollars	Few MHz	Medium to large	Hardware	Complete SoC
Virtual prototyping [17]	Small	Few thousand dollars	Few KHz	Small	Software	N/A
FPGA-based prototyping [11]	Medium to long	Few thousand dollars	Real time	Small	Hardware/software	Module level

**Table 2:** Benchmark description.

Sr. No.	Benchmark Name	Benchmark Type	No. of Components
1	CPU20	mono-cluster	50,460
2	CPU30	mono-cluster	65,620
3	CPU50	mono-cluster	85,260
4	CPU125	mono-cluster	120,526
5	AES	multi-cluster	90,680
6	CPU2X2X1	multi-cluster	93,654
7	CPU2X2X2	multi-cluster	105,426
8	CPU2X2X3	multi-cluster	119,256
9	CPU2X2X4	multi-cluster	133,459
10	CPU2X2X5	multi-cluster	368,125
11	CPU2X2X6	multi-cluster	380,783
12	CPU2X2X7	multi-cluster	395,487
13	CPU2X2X8	multi-cluster	1,296,458
14	CPU4X4X2	multi-cluster	1,319,258

**Table 3:** Cut-net results of two partitioning approaches.

Sr. No.	Benchmark	Cut Nets			Partitioning
	Name	Biterminal	Multiterminal	Total	Approach
1	CPU20	7561	2940	10,501	Multilevel
2	CPU30	8886	3456	12342	-
3	CPU50	11,933	4640	16,573	-
4	CPU125	15,447	6007	21,454	-
5	AES	7700	2995	10,695	-
6	CPU2X2X1	6304	2452	8756	Hierarchical
7	CPU2X2X2	6953	2704	9657	-
8	CPU2X2X3	7371	2867	10,238	-
9	CPU2X2X4	8891	3458	12,349	-
10	CPU2X2X5	10,013	3894	13,907	-
11	CPU2X2X6	10,315	4011	14,326	-
12	CPU2X2X7	11,113	4322	15,435	-
13	CPU2X2X8	18,487	7189	25,676	-
14	CPU4X4X2	21,835	8492	30,327	-

**Table 4:** MUX ratio comparison of different inter-FPGA routing approaches.

Sr. No.	Benchmark	Routing Approach			
	Name	RD	TD	RLM	RLNM
1	CPU20	2	2	2	3
2	CPU30	3	2	2	3
3	CPU50	5	5	5	5
4	CPU125	16	16	15	16
5	AES	7	7	6	8
6	CPU2X2X1	5	4	4	5
7	CPU2X2X2	5	4	4	6
8	CPU2X2X3	6	5	5	6
9	CPU2X2X4	7	6	6	7
10	CPU2X2X5	8	7	7	8
11	CPU2X2X6	8	8	8	9
12	CPU2X2X7	9	8	9	9
13	CPU2X2X8	12	10	10	13
14	CPU4X4X2	16	15	15	16

**Table 5:** Frequency comparison of different inter-FPGA routing approaches.

Sr. No.	Benchmark	Routing Approach			
	Name	RD	TD	RLM	RLNM
1	CPU20	18	20.9	20.9	15.7
2	CPU30	18	20.85	20.85	18
3	CPU50	14	14	14	14
4	CPU125	6.3	6.63	7	6.3
5	AES	11.5	11.5	12.6	11
6	CPU2X2X1	13.9	14	14	13.9
7	CPU2X2X2	12.6	14	14	12
8	CPU2X2X3	12.6	12.6	12.6	12.6
9	CPU2X2X4	11.3	12.6	12.6	11.3
10	CPU2X2X5	9.6	10.5	10.5	9.6
11	CPU2X2X6	9.7	10.5	10.5	9
12	CPU2X2X7	9.7	9.7	9	9.7
13	CPU2X2X8	7.9	8.4	8.4	7.4
14	CPU4X4X2	6	6.6	6.6	6

## Data Availability

Data are contained within the article.
